# Identification and Characterization of Roseltide, a Knottin-type Neutrophil Elastase Inhibitor Derived from *Hibiscus sabdariffa*

**DOI:** 10.1038/srep39401

**Published:** 2016-12-19

**Authors:** Shining Loo, Antony Kam, Tianshu Xiao, Giang K. T. Nguyen, Chuan Fa Liu, James P. Tam

**Affiliations:** 1 School of Biological Sciences, Nanyang Technological University, 60 Nanyang Drive, 637551, Singapore

## Abstract

Plant knottins are of therapeutic interest due to their high metabolic stability and inhibitory activity against proteinases involved in human diseases. The only knottin-type proteinase inhibitor against porcine pancreatic elastase was first identified from the squash family in 1989. Here, we report the identification and characterization of a knottin-type human neutrophil elastase inhibitor from *Hibiscus sabdariffa* of the Malvaceae family. Combining proteomic and transcriptomic methods, we identified a panel of novel cysteine-rich peptides, roseltides (rT1-rT8), which range from 27 to 39 residues with six conserved cysteine residues. The 27-residue roseltide rT1 contains a cysteine spacing and amino acid sequence that is different from the squash knottin-type elastase inhibitor. NMR analysis demonstrated that roseltide rT1 adopts a cystine-knot fold. Transcriptome analyses suggested that roseltides are bioprocessed by asparagine endopeptidases from a three-domain precursor. The cystine-knot structure of roseltide rT1 confers its high resistance against degradation by endopeptidases, 0.2 N HCl, and human serum. Roseltide rT1 was shown to inhibit human neutrophil elastase using enzymatic and pull-down assays. Additionally, roseltide rT1 ameliorates neutrophil elastase-stimulated cAMP accumulation *in vitro*. Taken together, our findings demonstrate that roseltide rT1 is a novel knottin-type neutrophil elastase inhibitor with therapeutic potential for neutrophil elastase associated diseases.

*Hibiscus sabdariffa*, commonly known as roselle or red sorrel, is a shrub belonging to the Malvaceae family. The roselle plant is native to Central and West Africa and is cultivated extensively in subtropical and tropical regions, such as in India and Southeast Asia^1^. Its rose-color petals are used in drinks as food coloring and its fruit is used to make fruit preservatives, syrups, and jams. As a traditional medicine, roselle is primarily used as a remedy for coughs^2^ but is also used as anti-hypertensive^3^,^4^, anti-hyperlipidemia^5^,^6^, anti-nociceptive, anti-pyretic, anti-inflammatory^7^, and diuretic agents^8^,^9^. Previous studies have shown that the roselle calyx extract contains proteinase inhibitors against angiotensin I converting enzyme, elastase, trypsin, and alpha-chymotrypsin^10^; however, the chemical nature of these proteinase inhibitors remains unexplored. To date, the known bioactive compounds of *Hibiscus sabdariffa* are limited to metabolites, including anthocyanins, flavonoids, saponins, tannins, phenols, glycosides, and alkaloids^11^,^12^,^13^.

Plant-derived peptides represent a promising group of natural products in drug discovery, as they fulfill the neglected chemical space between small-molecule metabolites (<1 kDa) and proteins (>8 kDa); however, these peptides have not received much attention as putative active compounds in medicinal plants and in drug development. Within the chemical space of 2–8 kDa, cysteine-rich peptides (CRPs), which possess multiple disulfide bridges to enhance both structural and physical stabilities, fulfill the criteria of putative bioactive peptides in medicinal plants^14^. Cysteine-rich peptides are classified into families primarily based on their cysteine motifs^15^,^16^,^17^. However, plant knottins are characterized structurally by their cystine-knot arrangement and their bioactivity as inhibitors. In particular, some knottins function as proteinase inhibitors against carboxypeptidase^18^, trypsin^19^,^20^, amylase^21^,^22^,^23^, and elastase^24^,^25^. Elastase inhibitors are of therapeutic interest since human neutrophil elastase is involved in several inflammatory diseases, including chronic obstructive pulmonary diseases (COPD), asthma, and cystic fibrosis^26^. Elastases are a class of serine proteinases that enzymatically degrade insoluble, highly cross-linked elastins. Serine proteinases have been reported to cleave and activate proteinase-activated receptors (PARs), a family of G protein-coupled receptors (GPCRs)^27^. Neutrophil elastase is recognized as a biased agonist of PAR2 and causes inflammation and pain^28^,^29^, as well as cough exaggeration^30^. Unlike trypsin inhibitors, elastase inhibitors appear to be a rarity. The only plant knottin-type elastase inhibitor was isolated from the squash family of *Mormodica charantia*^24^,^25^. The full length sequence MCEI-IV was identified in 1995, but three of its N-terminal truncated analogs MCEI-I to MCEI-III was first isolated in 1989. All four elastase inhibitors have been shown to inhibit porcine pancreatic elastases; however, an understanding of their inhibitory effect on human neutrophil elastases remains unknown.

In a mass-spectrometry-driven profiling program for CRPs in medicinal plants, we found a cluster of CRPs in the range of 2 to 4 kDa from the aqueous extracts of roselle plants. Herein, we report the identification and characterization of a knottin-type neutrophil elastase inhibitor (KNEI), roseltide rT1, from the medicinal plant *Hibiscus sabdariffa* of the Malvaceae family. Using a combination of proteomic and transcriptomic methods, we identified a panel of cysteine-rich peptides, collectively named roseltides (rT1-rT8). Transcriptomic analysis demonstrated that roseltides are bioprocessed from a three-domain precursor with Asn at the bioprocessing site to yield a mature roseltide. The prototypic and smallest member of roseltides, the 27-residue roseltide rT1, was shown to be a human neutrophil elastase inhibitor. Roseltide rT1 has a cystine-knot disulfide connectivity with a cysteine spacing that differs from the squash knottin-type elastase inhibitors. Taken together, our findings report the discovery and characterization of roseltide rT1, a novel plant-derived knottin-type neutrophil elastase inhibitor.

## Materials and Methods

### Materials

All chemicals and solvents, unless otherwise mentioned, were purchased from Sigma-Aldrich, USA and ThermoFisher Scientific, USA. pGlosensor-20F vector was purchased from Promega, SG. pCMV6-XL5 encoding PAR2 receptor (NM 005242.3) was purchased from Origene, USA. Anti-PAR2 antibody (SAM11) Alexa Fluor 647 was purchased from Santa Cruz Biotechnology, USA.

### Plant materials

*Hibiscus sabdariffa* were collected from the Nanyang Community Herb Garden, Nanyang Technological University, Singapore (courtesy of Mr. Ng Kim Chuan). The authenticity of samples was determined taxonomically by Lee, S. and Lam, H.J. of the Singapore Botanic Gardens and voucher specimens were deposited at the Singapore Herbarium in Singapore Botanic Gardens (code number: SING 2015-144). Dried calyces of *Hibiscus sabdariffa* were purchased from Hung Soon Medical Trading Pte Ltd, Singapore.

### Screening and profiling

Fresh plant parts of *Hibiscus sabdariffa* were extracted with water for 15 min at room temperature in 1:10 ratio. The aqueous extract was vortexed vigorously and centrifuged at 16,000 × g for 5 min at 4 °C and subjected to flash chromatography by C18 solid phase extraction (SPE) columns (Waters, USA). The fractions were eluted with 60% ethanol/0.01% trifluoroacetic acid (TFA) and analyzed by matrix-assisted laser desorption/ionization-time of flight mass spectrometry (MALDI-TOF MS) (AB SCIEX 4700 MALDI-TOF/TOF).

### Scale-up extraction and purification of Roseltide rT1

Dried calyces (1 kg) of *Hibiscus sabdariffa* were extracted for 15 min with water and centrifuged at 9,000 rpm for 10 min at 4 °C (Beckman Coulter, USA) and the supernatant was filtered through 1 μM pore size glass fiber filter paper (Sartorius, Singapore). The filtrate was then loaded onto a C18 flash column (Grace Davison, US) and eluted with 60% ethanol/0.01% TFA. The eluted fractions were then loaded onto an SP Sepharose resin column (GE Healthcare, UK), eluted with 1 M NaCl (pH 3.0), and followed by ultrafiltration (ViVaflow 200, 2000 MWCO hydrostat). Further purification was performed by reversed-phase high performance liquid chromatography (RP-HPLC) (Shimadzu, Japan). A linear gradient of mobile phase A (0.05% TFA/H_2_O) and mobile phase B (0.05% TFA/ACN) was used on the C18 column (250 × 22 mm, 5 μm, 300 Å) (Grace Davison, US). MALDI-TOF MS was used to identify the presence of roseltide rT1 in the eluted fractions. The eluted fractions were lyophilized for storage at room temperature.

### S-reduction and S-alkylation

Purified roseltide rT1 (1 mg/mL) was *S*-reduced by 10 mM dithiothreitol (DTT) in ammonium bicarbonate buffer (25 mM) pH 8 at 37 °C for 30 min, followed by *S*-alkylation with 60 mM of iodoacetamide (IAM) at 37 °C for 45 min. MALDI-TOF MS was used to confirm the mass shift after *S*-reduction and *S*-alkylation.

### De novo peptide sequencing

*S*-alkylated roseltide rT1 (1 mg/mL) were digested with trypsin or chymotrypsin in 5:1 (v/v) ratio in ammonium bicarbonate buffer (25 mM), pH 8 at 37 °C for 10 min. The digested peptide fragments were then analyzed by MALDI-TOF MS followed by MS/MS (AB SCIEX 4700 MALDI-TOF/TOF). *De novo* peptide sequencing was performed using the *b*-ions and *y*-ions.

### Total RNA isolation and next generation transcriptome sequencing

RNA isolation from fresh *Hibiscus sabdariffa* calyces was performed based on the protocol by Djami-Tchatchou and Straker (2011) using CTAB extraction buffer (2% cetyltrimethylammonium bromide, 2% polyvinylpyrrolidone, 100 mM Tris-HCl (pH 8.0), 2 mM EDTA, 2 M NaCl, 2% 2-mercapthoethanol)^31^. RNA library construction was performed using 1 μg of total RNA (RIN value >7.0) by Illumina TruSeq mRNA Sample Prep kit (Illumina, Inc., San Diego, CA, USA). Briefly, poly-A containing mRNA molecules were purified using poly-T-attached magnetic beads. Following purification, mRNA fragmentation was performed using divalent cations under elevated temperature. RNA fragments were reverse-transcribed into first strand cDNA using SuperScript II reverse transcriptase (Invitrogen) and random primers, followed by second strand cDNA synthesis using DNA Polymerase I and RNase H. These cDNA fragments were subjected to end repair process, the addition of a single ‘A’ base, and ligation of the indexing adapters. The products were then purified and enriched using PCR to create the final cDNA library. The libraries were quantified using qPCR according to the qPCR Quantification Protocol Guide (KAPA Library Quantification kits for Illumina Sequencing platforms) and qualified using the TapeStation D1000 ScreenTape (Agilent Technologies, Waldbronn, Germany). Indexed libraries were sequenced using the HiSeq2500 platform (Illumina, San Diego, USA) by Macrogen Inc. (Korea).

### Peptide mapping using a peptidomic approach method

Identification of putative roseltide sequences and confirmation of sequence of roseltide rT1 were performed using methods described by Serra et al (2016)^32^. One-pot reduction and alkylation were performed on the fractionated peptides. The peptide fractions were subjected to 30 mM dithiothreitol (DTT) and 60 mM bromoethylamine (BrEA) in 0.2 M Tris-HCl buffer (pH 8.6) at 55 °C for 60 min and the reaction was quenched using HCl. The reduced alkylated peptide samples were desalted using a C18 Sep-pack column (Waters, USA) and dried using SpeedVac (without heating). After re-dissolving the peptide solutions in H2O, they were analyzed using LC-MS/MS performed using an Orbitrap Elite mass spectrometer (Thermo Scientific Inc., Bremen, Germany) coupled with a Dionex UltiMate 3000 UHPLC system (Thermo Scientific Inc., Bremen, Germany). Samples were sprayed using a Michrom’s Thermo CaptiveSpray nanoelectrospray ion source (Bruker-Michrom Inc, Auburn, USA) and separation was perfored using a reverse phase Acclaim PepMap RSL column (75  μm ID  ×  15 cm, 2 μm particles; Thermo Scientific). The mobile phase was 0.1% formic acid (FA) as eluent A and 90% ACN 0.1% FA as eluent B, with a flow rate of 0.3 μL/min. A 60 min gradient was used for the elution as follows: 3% B for 1 min, 3–35% B over 47 min, 35–50% B over 4 min, 50–80% B over 6 s, 80% for 78 s; then, it was reverted to the initial state over 6 s and maintained for 6.5 min.

The Thermo Scientific Orbitrap Elite mass spectrometer was set to positive ion mode using LTQ Tune Plus software (Thermo Scientific Inc., Bremen, Germany) for data acquisition, alternating between a Full FT-MS (350–3000 m/z, resolution 60.000, with 1 μscan per spectrum) and a FT-MS/MS scan applying 65, 80, and 95 ms ETD activation times (150–2000 m/z, resolution 30.000, with 2 μscan averaged per MS/MS spectrum). The 3 most intense precursors with a charge >2+ were isolated with a 2 Da mass isolation window and fragmented. The automatic gain control (AGC) for Full MS and MS^2^ was set to 1  ×  10^6^ and the reagent AGC was 5  ×  10^5^. Data analysis was performed using PEAKS studio (version 7.0, Bioinformatics Solutions, Waterloo, Canada), where 10 ppm MS and 0.05 Da MS/MS tolerances were applied. A false discovery rate of 0.1% was applied to accept the sequences.

### Acid and proteolytic stability of roseltide rT1

#### Acid stability

0.1 M of purified roseltide rT1 was dissolved in 0.2 M HCl or phosphate buffered saline (PBS) (control) and incubated at 37 °C. Samples were collected at various time points (0, 15, 30, 45, 60 and 120 min).

#### Enzymatic stability

0.1 M of purified roseltide rT1 was dissolved in 100 mM Tris buffer (pH 8) and incubated with trypsin (Catalog number: T1426, Roche Applied Science, US) in a 50:1 (w/v) ratio at 37 °C. 0.1 M of purified roseltide rT1 was dissolved in 0.2 M HCl and incubated with pepsin (Catalog number: 03117901001, Roche Applied Science, US) in a 50:1 (w/v) ratio at 37 °C. Trypsin substrate and DALK (KRPPGFSPL-NH2) were used as controls, respectively. Samples were collected at various time points (0, 15, 30, 45, 60 and 120 min).

#### Human serum stability

0.1 M of purified roseltide rT1 was prepared in 25% human serum in DMEM medium without phenol red. The test samples were incubated at 37 °C. DALK was used as a positive control. Samples were collected at various time points (0, 24 and 48 h). The collected samples were subjected to protein precipitation with 100% ethanol and centrifugation at 180,000 g for 5 min at 4 °C. The supernatant was collected for analysis.

#### Analysis for stability assays

All collected samples from various stability assays were analyzed by RP-UHPLC with a linear gradient of mobile phase A (0.05% TFA/H_2_O) and mobile phase B (0.05% TFA/ACN) on C18 column (3.6 μm particle size; 2.1 mm internal diameter; 10 cm length) (Phenomenex, US). The resulting peaks were collected and the identified by MALDI-TOF MS. The results were expressed as percentage of initial concentration using the peak area of the UHPLC profile.

### Human neutrophil elastase inhibition assay

Human neutrophil elastase (HNE) activity was determined by measuring the release of p-nitroanilide at 405 nm at 37 °C using N-Methoxysuccinyl-Ala-Ala-Pro-Val p-nitroanilide as substrates. Purified roseltide rT1 was incubated with 1.75 U/mL HNE and 0.6 mM substrate in 100 mM Tris buffer (pH 8.0) at 37 °C; absorbance was measured continuously for 1 h. A synthetic elastase inhibitor, N-methoxysuccinyl-Ala-Ala-Pro-Val-chloromethyl ketone, was used as a positive control. The results were presented as normalized initial velocity.

### Peptide biotinylation and pull-down assay

Purified roseltide rT1 was biotinylated with EZ-Link NHS-LC-biotin (Thermo Fisher Scientific, US) in 100 mM phosphate buffer at a pH of 7.8. Biotinylation was carried out at room temperature for 2 h and the biotinylated peptide was then identified and purified by MALDI-TOF MS and RP-HPLC.

Pull-down assay was performed using NeutrAvidin UltraLink Resin (Thermo Fisher Scientific, US). Briefly, the resin was washed with PBS three times and incubated with biotinylated roseltide rT1 or biotin (control) at room temperature with rotation for 1 h. The resin was washed again with excess PBS six times before incubation with HNE overnight at 4 °C with rotation. The resin was washed with excess PBS for six times and dissociation was performed by the addition of 6x loading dye with 2-mercaptoethanol and was heated at 85 °C for 10 min. The samples were then resolved in 12% SDS-PAGE for 1.5 h. Silver staining was performed for protein visualization.

### Cell culture

Huh7 (human liver carcinoma cells), A549 (human lung adenocarcinoma epithelial cells) cells, and Chinese Hamster Ovary-K1 cells (CHO-K1) were cultured in Dulbecco’s Modified Eagle’s Medium (DMEM)/Ham’s F12 containing 15 mM HEPES and L-glutamine and supplemented with 10% fetal bovine serum, 100 U/mL of penicillin, and streptomycin. The cells were grown in a 5% CO_2_ humidified incubator at 37 °C. CHO-K1 cells were transfected with pGlosensor-20F plasmid by electroporation and selected using 500 μg/mL hygromycin. CHO-K1 cells stably expressing the pGlosensor-20F construct (cAMP-CHO) were then transfected with pCMV6-XL5 encoding PAR2 receptor (NM 005242.3) and selected using 500 μg/mL G418. Stable cell lines co-expressing both pGlosensor-20F and PAR2 receptor constructs (PAR2-cAMP-CHO) were maintained with 500 μg/mL hygromycin and 500 μg/mL G418.

### Flow cytometry analysis

PAR2-cAMP-CHO and cAMP-CHO cells were harvested and collected by centrifugation at 500 g for 5 min at 4 °C. The cell pellet was stained with the anti-PAR2 antibody (SAM11) Alexa Fluor 647 (1:100, Santa Cruz Biotechnology, USA) in serum-containing medium for 30 min on ice. The pellet was then washed three times and samples were analyzed by flow cytometry. 10,000 cells were analyzed using the BD LSRFortessa X-20 flow cytometer.

### Immunofluorescence analysis

PAR2-cAMP-CHO and cAMP-CHO cells were seeded on an 8-well chamber slide (ibidi, Germany). The slides were washed gently with PBS, fixed in 4% paraformaldehyde for 10 min, and permeabilized in PBS containing 0.25% triton X-100 for 15 min. The slides were blocked in PBS containing 3% BSA for 1 h and then incubated with anti-PAR2 antibody (SAM11) Alexa Fluor 647 (1:50, Santa Cruz Biotechnology, USA) for 1 h. After PBS washing, the slides were mounted with Fluoroshield containing DAPI (Sigma, USA) and observed under a Zeiss LSM 710 confocal microscope (ZEISS, Germany).

### Glosensor cAMP assay

Glosensor cAMP assays were performed according to the manufacturer’s instructions. Briefly, PAR2-cAMP-CHO cells were grown until they reached confluency in a white-walled, clear-bottom 96 well plate. The culture medium was replaced with 6% (v/v) Glosensor cAMP substrate in CO_2_-independent medium in the dark at room temperature for 1 h. Luminescent intensity was measured using a microplate reader (Tecan Infinite 200 Pro, Switzerland); the plate was pre-read for 30 s to establish basal luminescent level. Following treatment, luminescent levels were continuously monitored for 20 min. The results are presented as fold change relative to basal luminescent levels and were quantified using the area under the curve.

### Cell viability assay

Cell viability was measured using 3-(4,5-dimethylthiazolyl-2)-2,5-diphenyltetrazolium bromide (MTT) dye reduction assay. Briefly, cells were treated with roseltide rT1 or 0.1% triton X-100 (positive control) for 24 h. MTT (final concentration 0.5 mg/mL) was added and incubated for 3 h at 37 °C. Dimethyl sulfoxide was then added to dissolve the insoluble formazan crystal. The absorbance was measured at 550 nm using a microplate reader (Tecan Infinite 200 Pro, Switzerland).

### NMR spectroscopy and structure determination of roseltide rT1

A sample of roseltide rT1 for NMR spectroscopy was prepared by dissolving the lyophilized peptide in water containing 5% D_2_O at a final peptide concentration of 1.5 mM. For H/D exchange NMR experiment, the sample was dissolved in solution with 100% D_2_O immediately before the experiment. All NMR spectra were collected at a sample temperature of 298 K on a Bruker AVANCE II 600 MHz NMR spectrometer equipped with four RF channels and a 5 mm z-gradient TCI cryoprobe. Phase-sensitive two-dimensional ^1^H, ^1^H-TOCSY and NOESY spectra were recorded with a spectral width of 12 ppm. For water suppression, excitation sculpting with gradients was applied to all NMR experiments. TOCSY and NOESY spectra were obtained with mixing times of 80 ms and 200 ms respectively. The proton chemical shifts were referenced to external sodium 2,2-dimethyl-2-silapentane-5-sulfonate (DSS). All measurements were recorded with 2048 complex data points and zero-filled to 2048 × 512 data matrices. Time domain data in both dimensions were multiplied by a 90°-shifted squared sine bell window function prior to Fourier transformation. Baseline correction was applied with a fifth order polynomial. NMR data were acquired and processed by TopSpin (Bruker BioSpin). The NMR spectra were processed with NMRpipe^33^. Sequence-specific assignments were achieved with 2D TOCSY and NOESY and NOEs were performed using SPARKY^34^. Distance restraints were derived based on the intensities of NOE cross peaks, which were divided to three classes: strong, 1 < d ≤ 1.8; medium, 1.8 < d ≤ 3.4; weak, 3.4 < d ≤ 5. Hydrogen bond restraints were determined based on H/D exchange 1D NMR experiment, in which amide protons exchanged with solvent deuterium for 24 hours in 298 K. The hydrogen bond restraints were defined as: N-O, 0.8~3.3 Å; HN-O, 0.6~2.2 Å. Dihedral angle restraints were derived from the ^3^J_HN-Hα_ coupling constant measured in 1D ^1^H NMR spectrum. The backbone Φ angle was considered between -100° to -160° if the coupling constant was larger than 8 Hz. Three-dimensional structures were reconstructed using CNSsolve 1.3^35^. The 6 cysteines were assumed to form disulfide bonds in structure calculation. Structures were displayed with Chimera^36^ and Pymol^37^ and validated with the online server PDBsum^38^.

### In silico modeling

The *in silico* docking was performed using automatic protein-protein docking server ClusPro Version 2.0^39^,^40^. Both the NMR structure of roseltide rT1 and the crystal structure of HNE (PDB entry:1HNE)^41^ were uploaded to the server. It uses rigid body docking protocol and the model was generated based on electrostatic potentials.

### Statistical analyses

Statistical comparisons were performed using GraphPad Version 6.0d (USA). Data were analyzed using one-way analysis of variance (ANOVA) followed by Newman-Keuls *post hoc* tests. Data were expressed as mean ± S.E.M and *P* < 0.05 was considered to be statistically significant.

## Results

### Isolation of roseltide rT1 from Hibiscus sabdariffa

**Figure 1:**
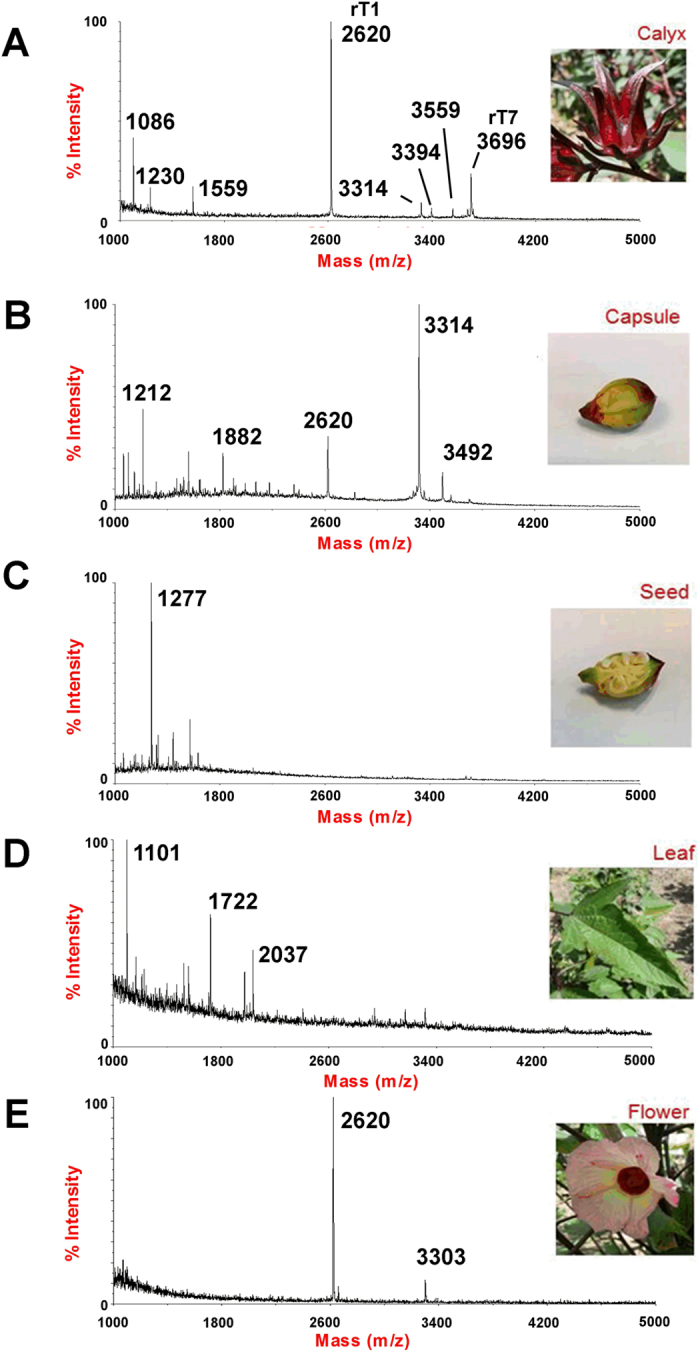
MS profiles of different plant parts of *Hibiscus sabdariffa*. (**A**) calyces, (**B**) capsule, (**C**) seed, (**D**) leaves, and (**E**) flower of *Hibiscus sabdariffa* were collected and profiled using Maldi-TOF MS.

Mass spectrometry profiling of the aqueous extracts of *Hibiscus sabdariffa* revealed the presence of a cluster of strong signals in the mass range of 2–4 kDa in the calyces, capsules and flowers (Fig. 1). We focused on one of the strongest signals of this cluster, the 2620 Da-peak which was found in the calyces, capsules and flower extracts. The 2620 Da-peak was shown to be a CRP with six cysteine residues based on a mass increase of 348 Da after *S*-reduction by dithiothreitol and *S*-alkylation by iodoacetamide (Supplementary S1). To characterize this CRP, a scale-up aqueous extraction was carried out using the calyces of *Hibiscus sabdariffa*. The crude aqueous extract was fractionated by C18 reversed-phase and strong cation-exchange flash chromatography, followed by ultrafiltration using a membrane with molecular weight cut-off of 2000 Da. The CRP-enriched fraction was further purified by RP-HPLC (Supplementary data S2), and the 2620-Da CRP was designated roseltide rT1. 6–8 mg of purified roseltide rT1 was obtained per Kg of dried calyces.

**Figure 2:**
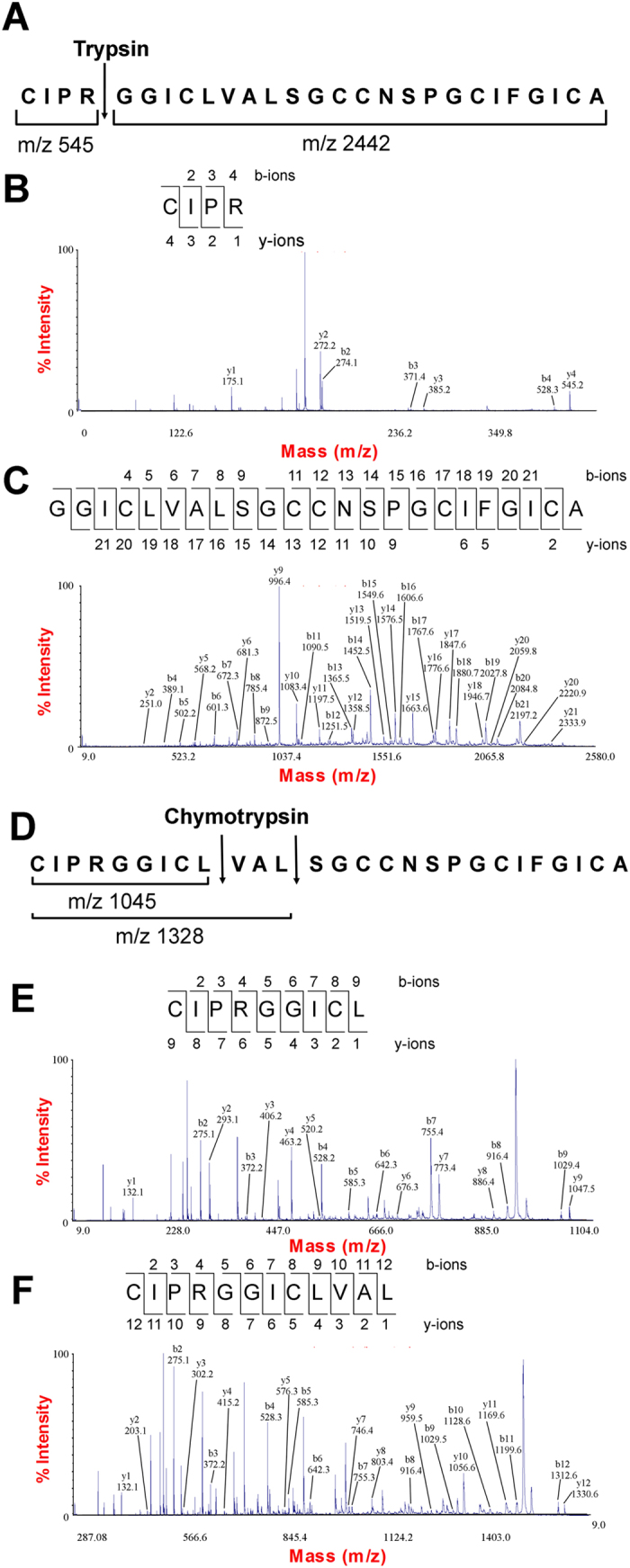
*De novo* sequencing of roseltide rT1. (**A**) *S*-alkylated roseltide rT1 was digested with trypsin, resulting in two tryptic fragments with the *m*/*z* of 545 and 2442; (**B**) MS/MS spectra of 545 Da fragment; (**C**) MS/MS spectra of 2442 Da fragment; (**D**) *S*-alkylated roseltide rT1 was digested with chymotrypsin at two sites, resulting in fragments with the *m*/*z* of 1045 and 1328. The third peptide fragment of *m*/*z* 1640 could not be detected.; (**E**) MS/MS spectra of 1045 Da fragment; (**F**) MS/MS spectra of 1328 Da fragment.

**Figure 3:**
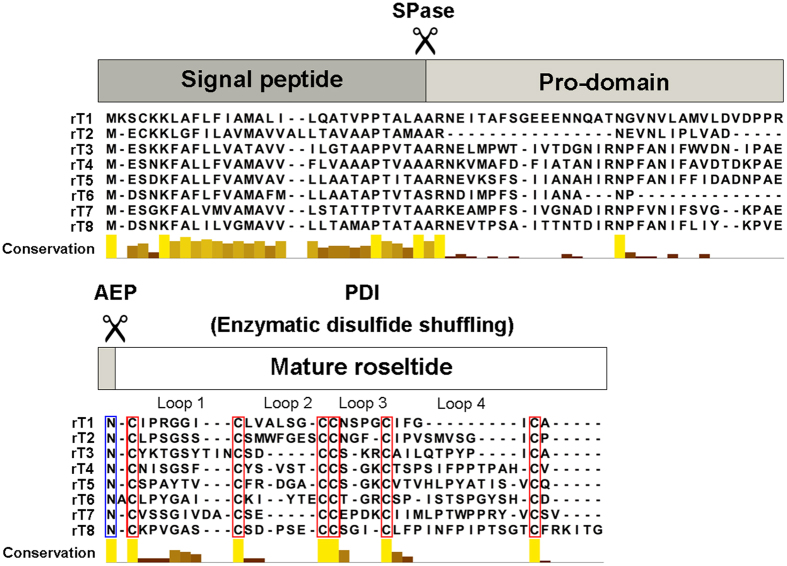
Roseltide transcripts from *Hibiscus sabdariffa*. The histogram depicts conservation among the putative amino acid sequences of roseltides by amino acid property grouping as determined by Jalview software. AEP: asparagine endopeptidase; PDI: protein disulfide isomerase; SPase: signal peptidase.

To determine the amino acid sequence of roseltide rT1, the purified roseltide rT1 was fully *S*-reduced and *S*-alkylated followed by digestion with either trypsin or chymotrypsin. The digested peptide fragments were analyzed by MALDI-TOF MS, followed by MS/MS sequencing. Analysis using the *b*-ions and *y*-ions revealed that roseltide rT1 is a 27-residue peptide with six cysteine residues (Fig. 2). The amino acid sequence of rT1 was confirmed by transcriptomic analysis. Transcriptome analyzes showed that roseltide rT1 was biosynthesized as a 90-residue precursor with three domains: a 28-residue N-terminal signal peptide, a 35-residue pro-domain and a 27-residue C-terminal mature peptide (Fig. 3). Using the asparaginyl endopeptidase cleavage site and the cysteine spacing pattern of roseltide rT1 in its transcriptomic database search, additional seven putative roseltide sequences (rT2-rT8) were identified (Fig. 3 & Table 1). Using a high-throughput peptidomic method for peptide sequencing developed by our laboratory^32^, we identified the presence of rT7 in the aqueous extract of *Hibiscus sabdariffa* calyces (Supplementary data S3).

### NMR structure of roseltide rT1

**Figure 4:**
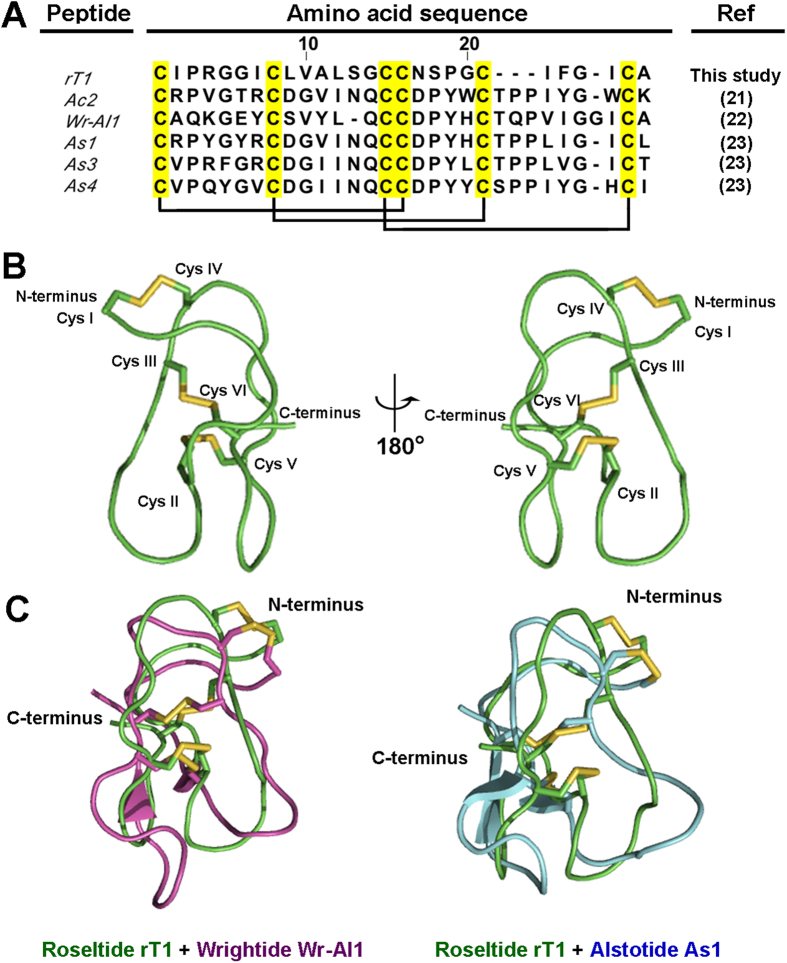
(**A**) Sequence alignment of CKAI with roseltide rT1; allotide Ac2 from *Allamanda cathartica*; wrightide Wr-Al1 from *Wrightia religiosa*; alstotides As1, As3 and As4 from *Alstonia scholaris* (**B**) Solution structure of roseltide rT1; (**C**) Superimposition of roseltide rT1 (green) on wrightide Wr-AI1 (PDB entry 2MAU) (purple) and alstotides As1 (PDB entry 2MM6) (blue).

The cysteine spacing of all eight roseltides contains a CC motif which provides a clue to their putative disulfide connectivity as a cystine-knot. Using peptide mapping, NMR, and X-ray crystallography, our laboratory has previously demonstrated in cystine-knot α-amylase inhibitors (CKAI) (Fig. 4A) that the six-Cys-containing CRPs having a CC motif of CIII and CIV such as roseltides are often arranged in a cystine-knot motif (Cys I-IV, Cys II-V and Cys III-VI)^21^,^22^,^23^.

To characterize the structural fold of roseltide rT1, the solution structure of rT1 was determined using 2D ^1^H, ^1^H- TOCSY and NOESY NMR spectra. The sequential assignment was done based on the NOE cross peaks between HN_i_ and Hα_i-1_ as well as the other side chain protons of residue i-1 (Supplementary data S4 and S5). When performing the assignment, we compared the NOESY spectrum and the TOCSY spectrum to differentiate the intra-residue and inter-residue NOEs. The amide proton of residue i should have NOE cross peaks with the side chain protons of the residue i-1. The pattern of the peaks in TOCSY of each amide proton stripe should correspond to its specific residue. Based on these strategies, the sequential assignment was completed. More than 95% of the peaks in the NOESY spectrum were assigned. H/D exchange NMR experiment indicated that the residue C26 and G5, I25 and I22 as well as I2 and C16 should be involved in hydrogen bonds, which was consistent with the NOE cross peaks between the amide protons of them accordingly. This observation further confirms the sequential assignment. Twenty structures with the lowest energy among 100 structures were generated by CNSsolve 1.3^35^ (Supplementary data S6). The 20 structures are highly converged, of which the backbone RMSD and the heavy atom RMSD are 0.71 ± 0.14 Å and 1.19 ± 0.26 Å respectively (Table 2). The ensemble of the 20 best structures has been deposited to Protein Data Bank (PDB) with the accession number 5GSF. The chemical shifts have been submitted to Biological Magnetic Resonance Bank (BMRB), of which the accession number is 26874 (Supplementary data S7).

The proton chemical shifts were uploaded to the online server CSI 3.0 (http://csi3.wishartlab.com/cgi-bin/index.php) to predict the secondary structure, indicating that only residue F23-C26 might be edge β strand while the other residues were coil^42^. The structure of rT1 generated by simulated annealing contains no α helix or β strand. The two prolines both adopt trans form, which are supported by the NOE cross peak between Hδ_i_ and HN_i-1_ of the proline and the previous residue respectively. The disulfide bonds Cys8-Cys21 and Cys15-Cys26 cross in the center of rT1. The disulfide bond Cys1-Cys16 makes the N terminus bend (Fig. 4B).

Further evidence supporting the Cys1-Cys16, Cys8-Cys21 and Cys15-Cys26 disulfide linkages were observed from the Hβ-Hβ NOE cross peaks (Supplementary data S8). To confirm the disulfide connectivity, all the six cysteines were assumed reduced in the simulated annealing by CNSsolve 1.3^35^. The 100 structures were highly converged. The backbone RMSD and the heavy atom RMSD of the 20 best structures are 0.73 ± 0.27 Å and 1.25 ± 0.26 Å respectively. The averaged energy of the 20 best structures is similar to that of the 20 best structures with the 3 disulfide bonds imposed (Cys1-Cys16, Cys8-Cys21 and Cys15-Cys26). The structures generated with and without the disulfide bond imposed are very similar, except for the sidechains of the six cysteines (Supplementary data S6). Moreover, another 14 disulfide patterns were assumed in structure calculations respectively. The average energy of the 20 best structures for each combination was compared with the one with the pattern: Cys1-Cys16, Cys8-Cys21 and Cys15-Cys26. The disulfide pattern of: Cys1-Cys16, Cys8-Cys21 and Cys15–26 has the lowest average energy among the 15 combinations (Supplementary data S9). These results strongly suggest that the disulfide connectivity of roseltide rT1 exists in a cystine-knot motif.

Protein tertiary structure comparison was conducted using the SuperPose software Version 1.0^43^ for the wrightide Wr-AI1 (PDB entry: 2MAU)^22^ and alstotide As1 (PDB entry: 2MM6)^23^ which displays similar cystine-knot fold (Fig. 4C). The RMSD values between the superimposed structure of wrightide Wr-AI1 and roseltide rT1 were 0.515 Å and 1.036 Å for all Cα and heavy atoms, respectively. The RMSD values between the superimposed structures of alstotide As1 and roseltide rT1 were 0.534 Å and 1.003 Å for all Cα and heavy atoms, respectively. Based on the electrostatic potential surface of roseltide rT1, a negatively-charged region was observed. This is created by the side-chain of Arg4 residue which is positioned outwards.

### Acid and proteolytic stability of roseltide rT1

**Figure 5:**
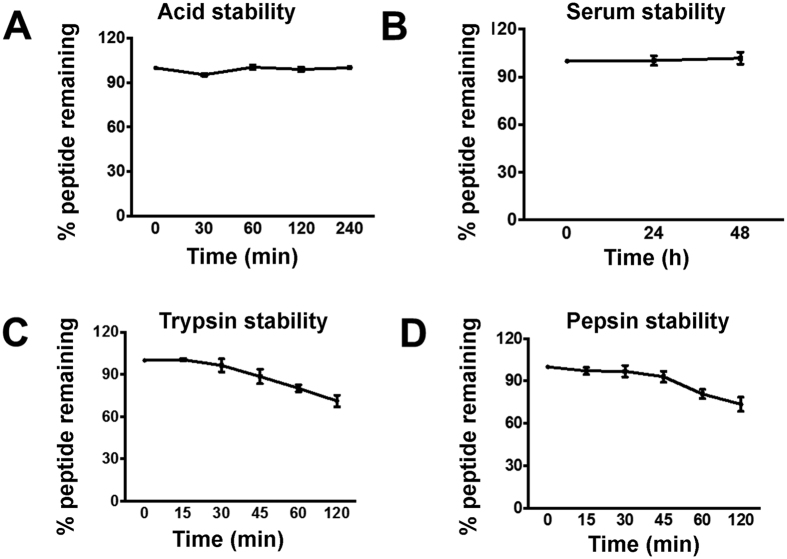
(**A**) Acid, (**B**) human serum, (**C**) trypsin, and (**D**) pepsin stability of roseltide rT1. All results were expressed as mean ± S.E.M. (n = 3).

Intact CRPs are highly cross-linked by multiple disulfide bridges which confers their high stability. To determine the acid and proteolytic stability of roseltide rT1, roseltide rT1 was incubated in 0.2 N HCl, or with proteinases (trypsin or pepsin), or in 25% human serum. The results demonstrated that roseltide rT1 was resistant against acid, proteinase and human serum-mediated degradation (Fig. 5).

### Roseltide rT1 is not cytotoxic

**Figure 6:**
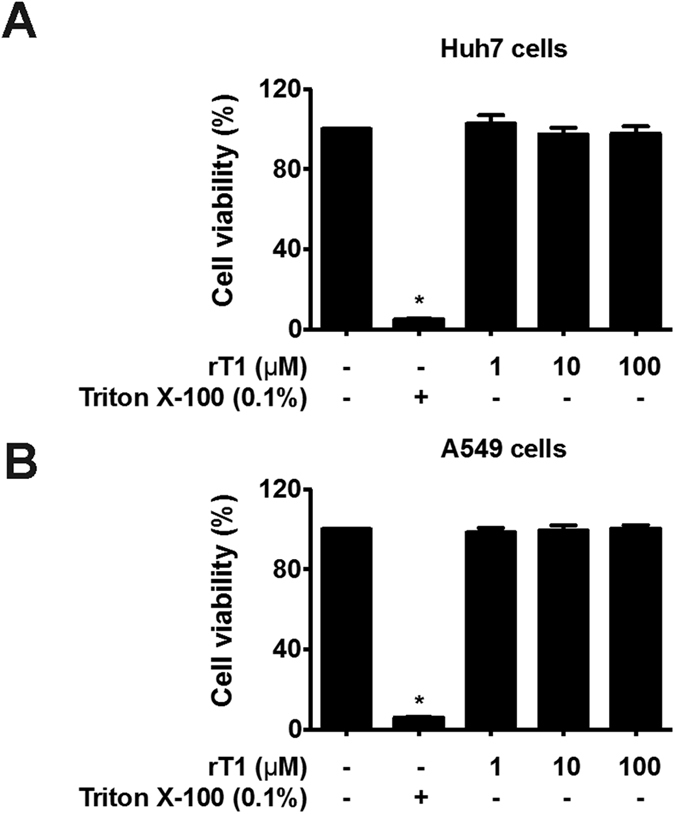
Roseltide rT1 does not show cytotoxic activities. Effects of roseltide rT1 on (**A**) Huh7 and (**B**) A549 cells. All results were expressed as mean ± S.E.M. (n = 3). *P < 0.05 compared to control group.

To determine the cytotoxicity of roseltide rT1, cell viability was measured by MTT assay. Treatment with roseltide rT1 of concentrations up to 100 μM for 24 h did not affect the viability of Huh7 (human liver carcinoma cells) and A549 (human lung adenocarcinoma epithelial cells) cells (Fig. 6).

### Roseltide rT1 inhibited human neutrophil elastase

**Figure 7:**
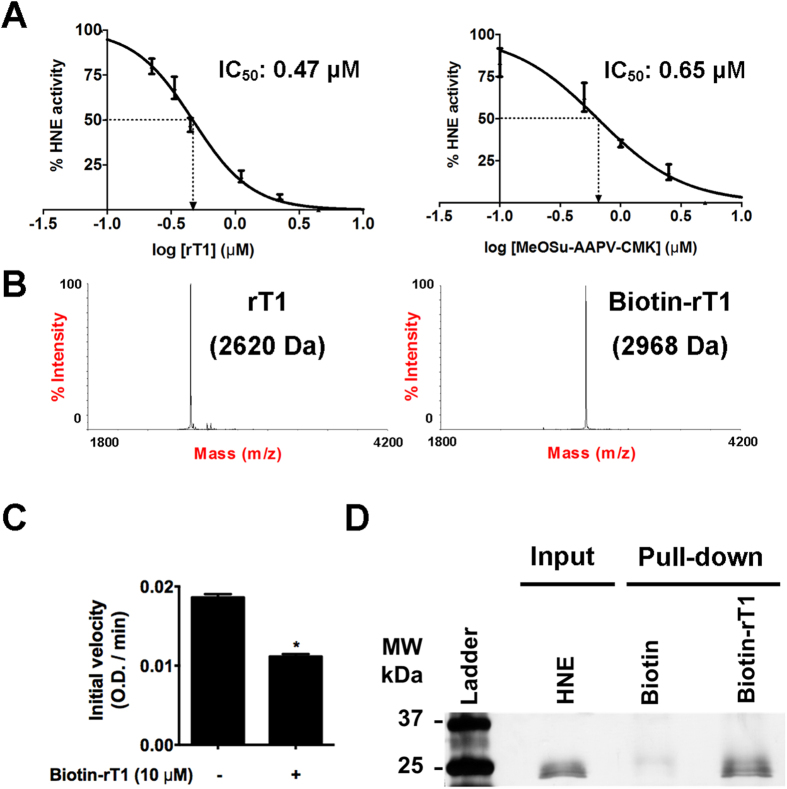
The effects of roseltide rT1 on human neutrophil elastase. (**A**) The effects of different concentrations of roseltide rT1 or synthetic elastase inhibitor, N-methoxysuccinyl-Ala-Ala-Pro-Val-chloromethyl ketone, on human neutrophil elastase (HNE) activity was measured at 405 nm at 37 °C using N-methoxysuccinyl-Ala-Ala-Pro-Val p-nitroanilide as a substrate. All results were expressed as mean ± S.E.M. (n = 3); (**B**) MS spectra showed biotinylation of roseltide rT1; (**C**) The effects of biotin-rT1 on HNE activity. All results were expressed as mean ± S.E.M. (n = 3). *P < 0.05 compared to control; (**D**) Silver-stained SDS-PAGE of the pull-down complex between HNE and biotin-rT1. The left-most lane shows a protein marker (Bio-rad, US); HNE-only lane: purified HNE only; biotin lane: purified HNE incubated with biotin and NeutrAvidin resin (control), biotin-rT1 lane: purified HNE incubated with biotin-rT1 and NeutrAvidin resin. The full-length gel image is provided in Supplementary data S11.

Previous studies have shown that the extract of *Hibiscus sabdariffa* calyces contains proteinase inhibitors against elastase^10^, however, the active compounds are yet to be reported. To characterize the biological activities of roseltide rT1, its effects on the enzymatic activities of human neutrophil elastase was examined. As shown in Fig. 7A, roseltide rT1 inhibited the enzymatic activities of human neutrophil elastase in a dose-dependent manner, with an IC_50_ of 0.47 μM. This was comparable to the activity of a synthetic elastase inhibitor (MeOSu-AAPV-CMK). Roseltide rT1 was also screened against trypsin and porcine pancreatic elastase without observable inhibitory effects up to 10 μM (data not shown).

### Roseltide rT1 showed protein interactions to human neutrophil elastase

Pull-down assays were performed to determine the binding of roseltide rT1 to human neutrophil elastase. Purified biotin-rT1 was characterized by MALDI-TOF MS and RP-HPLC (Fig. 7B). Following biotinylation, the MS profile showed an increase in mass from 2620 Da to 2960 Da. Biotin-rT1 inhibits human neutrophil elastase (Fig. 7C) and was able to pull-down human neutrophil elastase with a band of approximately 25 kDa (Fig. 7D). Pull-down assay was also performed using porcine pancreatic elastase however, biotin-rT1 does not show protein interactions with porcine pancreatic elastase (Supplementary data S10).

### Roseltide rT1 inhibited neutrophil elastase-induced cAMP accumulation

**Figure 8:**
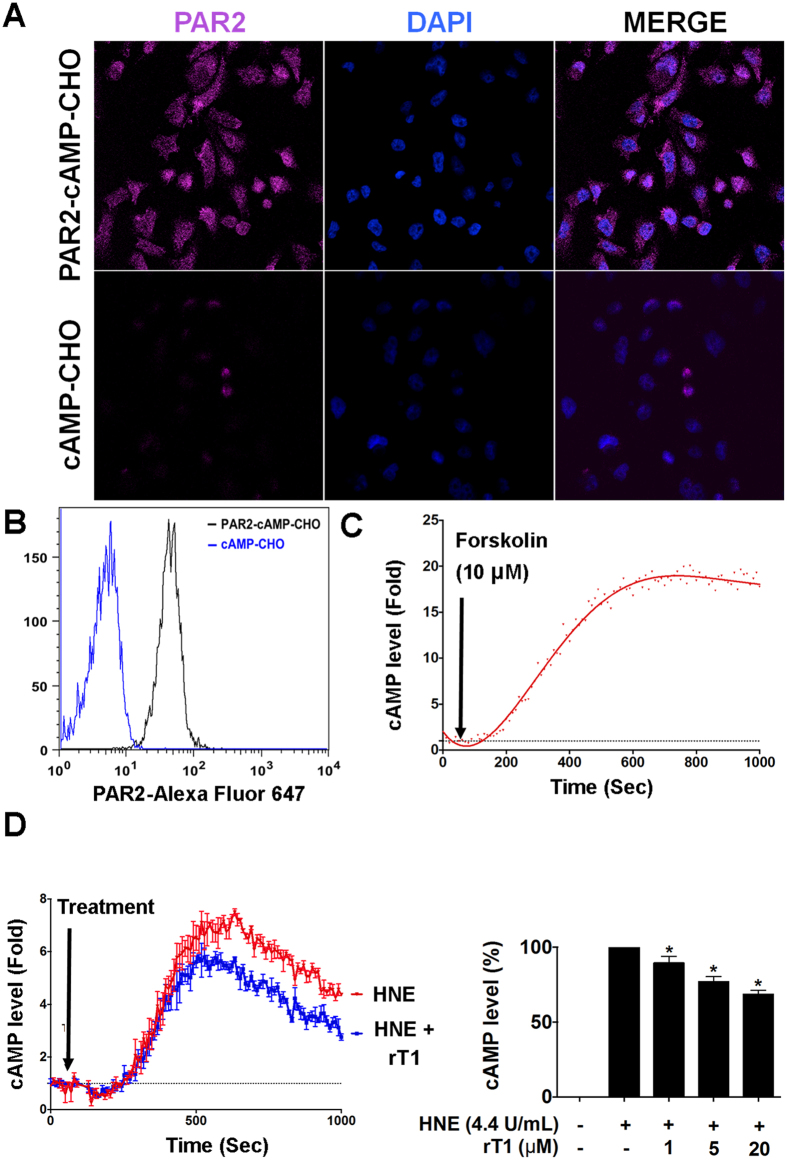
Roseltide rT1 ameliorates human neutrophil elastase (HNE)-stimulated cAMP accumulation in CHO-K1 cells co-expressed with PAR2 receptor and Glosensor cAMP biosensor constructs. Comparison of PAR2 receptor expressions in cAMP-CHO cells using (**A**) confocal microscopy and (**B**) flow cytometry; (**C**) Effects of cAMP activator Forskolin (10 μM) on cAMP accumulation in PAR2-cAMP-CHO cells; (**D**) Effects of HNE with or without roseltide rT1 on cAMP accumulation in PAR2-cAMP-CHO cells. All results were expressed as mean ± S.E.M. (n = 3). *P < 0.05 compared to HNE-treated group.

PAR2 is a GPCR responsible for the cellular effects of neutrophil elastase. To demonstrate the cellular effects of neutrophil elastase inhibition *in vitro*, the effects of roseltide rT1 on neutrophil elastase-stimulated cAMP accumulation were evaluated. In this study, CHO-K1 cells stably transfected with Glosensor cAMP biosensor (cAMP-CHO cells) was used to provide direct and real-time measurement of cAMP accumulation in live cells. The cAMP-CHO cells were further overexpressed with the gene of PAR2 receptor (PAR2-cAMP-CHO) cells. The expression levels of PAR2 were confirmed by flow cytometry and confocal microscopy while the function of cAMP biosensor was assessed using a cAMP activator, forskolin (10 μM) (Fig. 8A–C). Similar to previous findings, human neutrophil elastase stimulated intracellular cAMP accumulation in PAR2-cAMP-CHO cells. Co-incubation of human neutrophil elastase with roseltide rT1 significantly suppressed cAMP accumulation (Fig. 8D).

## Discussion

Plants are rich in proteinase inhibitors that serve as defense mechanisms against pests and pathogens^44^. This study employed a peptidomic approach to identify a panel of CRPs, roseltides (rT1-rT8), from the medicinal plant *Hibiscus sabdariffa*. Roseltide rT1, the smallest member of roseltide family, was identified as a knottin-type inhibitor against human neutrophil elastase. There has been only one reported knottin-type porcine elastase inhibitor (MCEI-IV) with three N-terminal truncated analogs (MCEI-I to MCEI-III) within the chemical space of 2–6 kDa from *Momodica charantia* of the squash family^24^,^25^. However, plant knottin-type elastase inhibitors are likely more prevalent than previously believed.

Transcriptomic and proteomic analyses showed that roseltides (rT1-rT8) contain six Cys residues with a cysteine spacing pattern of CX_6-8_CX_2-7_CCX_3-4_CX_4-13_C and four inter-cysteine loops (Fig. 3). Roseltides range in length from 27 to 39 amino acid residues with highly variable amino acid sequences in loops 2 and 4; for example, loop 4 is particularly variable in length, ranging from 4 residues in rT1 to 13 residues in rT8. It is important to note that the highly variable loop lengths of roseltides could be used as a grafting strategy for the engineering new bioactive peptides using roseltides as a scaffold^45^,^46^.

Transcriptome analyses demonstrated that all roseltide precursors possess a three-domain precursor of ER signal peptide, pro-domain, and mature domain. Sequence analyses showed that these precursors share similar signal peptide sequences and bioprocessing sites. This bioprocessing pathway was also observed in the precursor sequence of cystine-knot α-amylase inhibitors^21^,^22^,^23^. The N-terminal cleavage site for the mature peptide of roseltide rT1 occurs between Asn and Cys, suggesting that removal of the pro-domain is mediated by an asparaginyl endopeptidase, a vacuolar processing enzyme^47^.

The smallest and most abundant member of roseltides, roseltide rT1, is a single, positively charged peptide with six cysteine residues and a cysteine spacing pattern of CX_6_CX_6_CCX_4_CX_4_CX, a Cys-spacing motif which is prevalent in CKAI^21^,^22^,^23^. Of the 27 amino acids, seven are Ile/Val/Leu and a total of 85% of its residues are hydrophobic. The disulfide bridges of roseltide rT1, arranged in a cystine-knot motif, confer its high resistance against acid and proteolytic degradation. Many plant knottins have been identified as proteinase inhibitors, including the potato carboxypeptidase inhibitor (PCI), *Momordica cochinchinensis* trypsin inhibitor II (MCoTI-II), and *Ecballium elaterium* trypsin inhibitor II (EETI-II)^48^,^49^,^50^. Elastases are a class of serine proteinases that cleave C-terminal of small hydrophobic amino acids including Gly, Val, and Ala. Neutrophil elastase is one of the most potent elastases and has gained increased attention for its involvement in airway inflammatory diseases, particularly cystic fibrosis, asthma, COPD, and pulmonary emphysema^26^. Its underlying mechanisms have been attributed to the tissue-damaging, hypersecretory, and pro-inflammatory effects of neutrophil elastases^51^. Our results suggest that roseltide rT1 binds to human neutrophil elastase and inhibits its proteolytic activities. In comparison to the knottin-type elastase inhibitors from the squash inhibitors family (MCEI-I to MCEI-IV), there is no sequence homology to roseltide rT1 other than classification as a cystine-knot peptide^24^,^25^ (Table 3).

**Figure 9:**
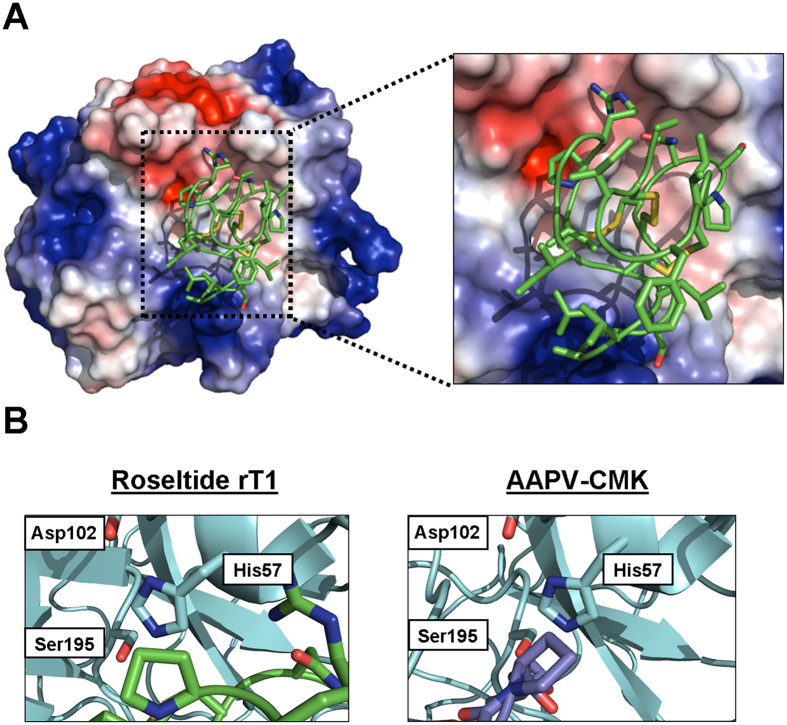
(**A**) Modeling the interaction between roseltide rT1 and the human neutrophil elastase (PDB entry: 1HNE) using the ClusPro Version 2.0 server. Blue: Negatively-charged, Red: Positively-charged and White: Neutral.; (**B**) Interactions between roseltide rT1 and peptide chloromethyl ketone inhibitor (AAPA-CMK) (PDB entry: 1HNE) with the catalytic triad of human neutrophil elastase formed by His57, Asp102, and Ser195.

Previous studies have demonstrated that the S1 pocket of elastases is narrowed by bulky side-chains, resulting in the recognition of small or medium-sized aliphatic P1 residues^52^. Compared to porcine pancreatic elastase (PPE), the S1 pocket of HNE is more flexible and is negatively charged^52^,^53^, which may explain the recognition of Lys or Arg at the P1 position of the inhibitor, leading to the selectivity of biotin-rT1 towards HNE (supplementary data S10). The S2 pocket of HNE is hydrophobic and can accept peptidyl inhibitors with Pro residue at its P2 position^54^,^55^,^56^. The S3 pocket of HNE is also hydrophobic and can tolerate small lipophilic residues, including Val and Ile, at the P3 position^54^; thus, it is likely that Ile-Pro-Arg in the inter-cysteine loop 1 of roseltide rT1 plays an important role in the interaction between the S1, S2, and S3 pockets of HNE. *In silico* modeling using the ClusPro server suggests that the Pro residue of roseltide rT1 interacts with the catalytic triad of HNE formed by His57, Asp102, and Ser195, which is similar to the known inhibitor AAPA-CMK (PDB entry: 1HNE) (Fig. 9)^39^,^40^,^41^,^57^. Additional studies are necessary to investigate key amino acid residues and the molecular mechanisms of the inhibitory activities of roseltide rT1 against neutrophil elastase using point mutation and X-ray crystallography.

Neutrophil elastase is a biased agonist of PAR2^28^. It cleaves the extracellular domain of PAR2 at Ser^67^ and Val^68^ to trigger its receptor activation and induces cAMP accumulation^58^. A previous study reported that PAR2 activation leads to TRPV1-triggered cough exaggeration in guinea pigs^30^. PAR2 activation by neutrophil elastase has also been shown to trigger airway hyperresponsiveness^59^, as well as TRPV4-mediated pain and inflammation^58^. Therefore, PAR2 activation plays a significant role in the deleterious effects of neutrophil elastase on inflammation. To investigate the cellular effects of neutrophil elastase inhibition by roseltide rT1, the intracellular level of cAMP was monitored in CHO-K1 cells co-expressed with PAR2 and cAMP biosensor constructs. The results showed that roseltide rT1 ameliorates neutrophil elastase-stimulated cAMP accumulation in PAR2-overexpressed cells. Hence, these findings suggest that metabolically-stable roseltide rT1 has therapeutic potential for neutrophil elastase-driven diseases, particularly in airway inflammatory diseases.

In conclusion, this study reports the identification of roseltides from *Hibiscus sabdariffa* and characterizes roseltide rT1 as a novel KNEI, 27 years after the report of the first squash knottin-type elastase inhibitor. Our findings highlight the therapeutic potential of roseltide rT1, a novel KNEI of the Malvaceae family, for neutrophil elastase-driven diseases, including COPD, asthma, and cystic fibrosis.

## Additional Information

**How to cite this article**: Loo, S. *et al*. Identification and Characterization of Roseltide, a Knottin-type Neutrophil Elastase Inhibitor Derived from *Hibiscus sabdariffa. Sci. Rep.* **6**, 39401; doi: 10.1038/srep39401 (2016).

**Publisher's note:** Springer Nature remains neutral with regard to jurisdictional claims in published maps and institutional affiliations.

## Supplementary Material

Supplementary Information

## Figures and Tables

**Table 1:**
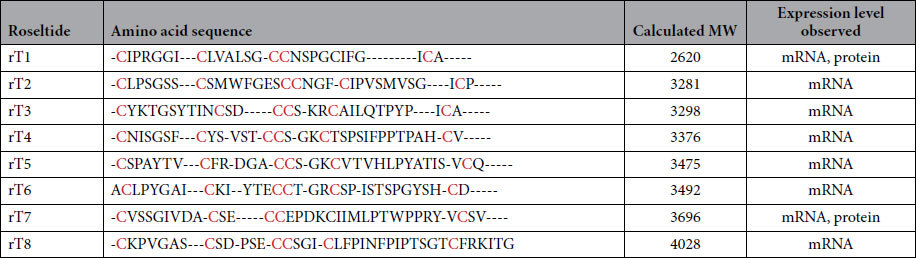
Putative amino acid sequences of roseltides.

**Table 2:** Parameters and restrains of structure calculation of roseltide rT1.

*Experimental Restraints and Structural Statistics of 20 Lowest-Energy Structures of GB4242 among the 100 Structures Generated by CNSsolve 1.3*		
*NMR Distance Restraints*	*431*	
* Intra-Residue NOE*	*97*	
* Sequential NOE (|i-j|=1*)	*127*	
* Medium-Range NOE (1<|i-j|≤5*)	*74*	
* Long-Range NOE (|i-j|>5*)	*133*	
* Hydrogen Bonds*	*6*	
*Dihedral Angle Restraints*	*8*	
*Structural Statistics (27 residues, C*^*1*^*-A*^*27*^)		
*Violations per Structure*		
* NOE Violation (Å*)	*0.025* *±* *0.002*	
* Torsion Angles (°*)	*0.064* *±* *0.04*	
*Ramachandran Plot Region (27 residues*)		
*Residues in Most Favored Regions*	*5*	*27.8%*
*Residues in Additional Allowed Regions*	*10*	*55.6%*
*Residues in Generously Allowed Regions*	*3*	*16.7%*
*Residues in Disallowed Regions*	*0*	*0%*
*Number of End-Residues (excl. Gly and Pro*)	*2*	
*Number of Glycine Residues*	*5*	
*Number of Proline Residues*	*2*	
*Mean RMSD from the Average Coordinates (27 residues, C*^*1*^*-A*^*27*^)		
* Backbone Atoms(Å*)	*0.71* *±* *0.14*	
* Heavy Atoms(Å*)	*1.19* *±* *0.26*	

**Table 3:**
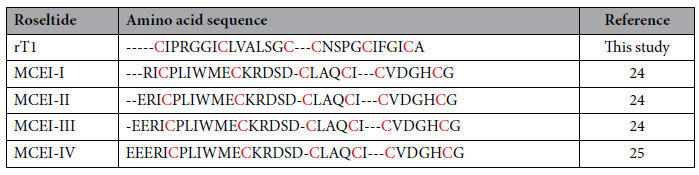
Sequence comparison between Roseltide rT1 and MCEI-I-IV.
