# Supplementary material for: Identification and Characterization of Roseltide, a Knottin-type Neutrophil Elastase Inhibitor Derived from Hibiscus sabdariffa
Source: Sci Rep. 2016 Dec 19;6:39401. doi: 10.1038/srep39401 (PMC5171801; doi:10.1038/srep39401)
Supplement: Supplementary Information [file srep39401-s1.doc]

# Identification and Characterization of Roseltide, a Knottin-type Neutrophil Elastase Inhibitor Derived from *Hibiscus sabdariffa*

**Shining Loo^a^, Antony Kam^a^, Tianshu Xiao, Giang K.T. Nguyen, Chuan Fa Liu, and James P. Tam^*^**

School of Biological Sciences, Nanyang Technological University, 60 Nanyang Drive, 637551, Singapore

________________________

^*^Corresponding author: Professor James P. Tam, School of Biological Sciences, Nanyang Technological University, 60 Nanyang Drive, 637551, Singapore

Email:JPTam@ntu.edu.sg

^a^These authors contributed equally to this work


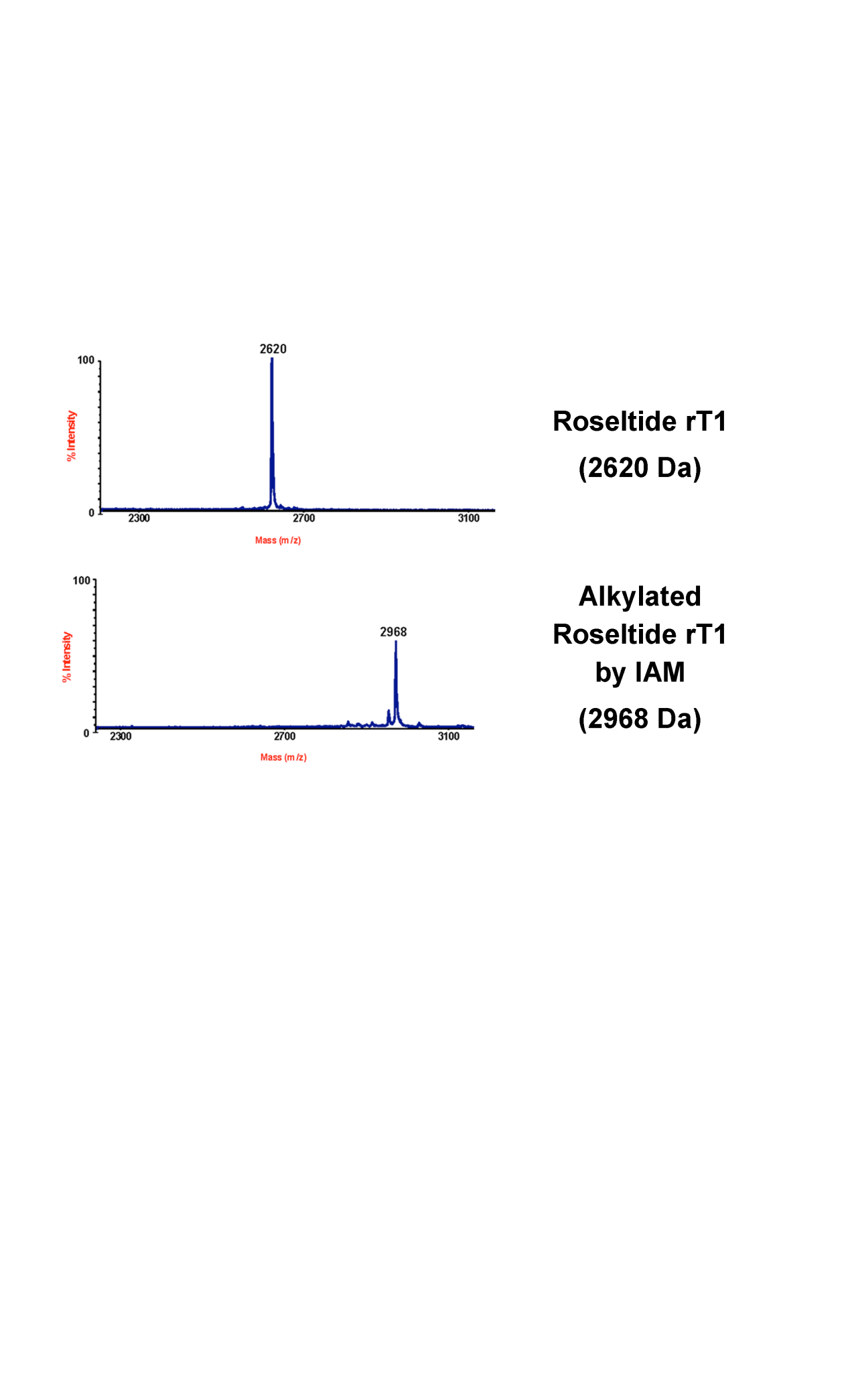


**Supplementary data S1.** Reduction and alkylation of Roseltide rT1


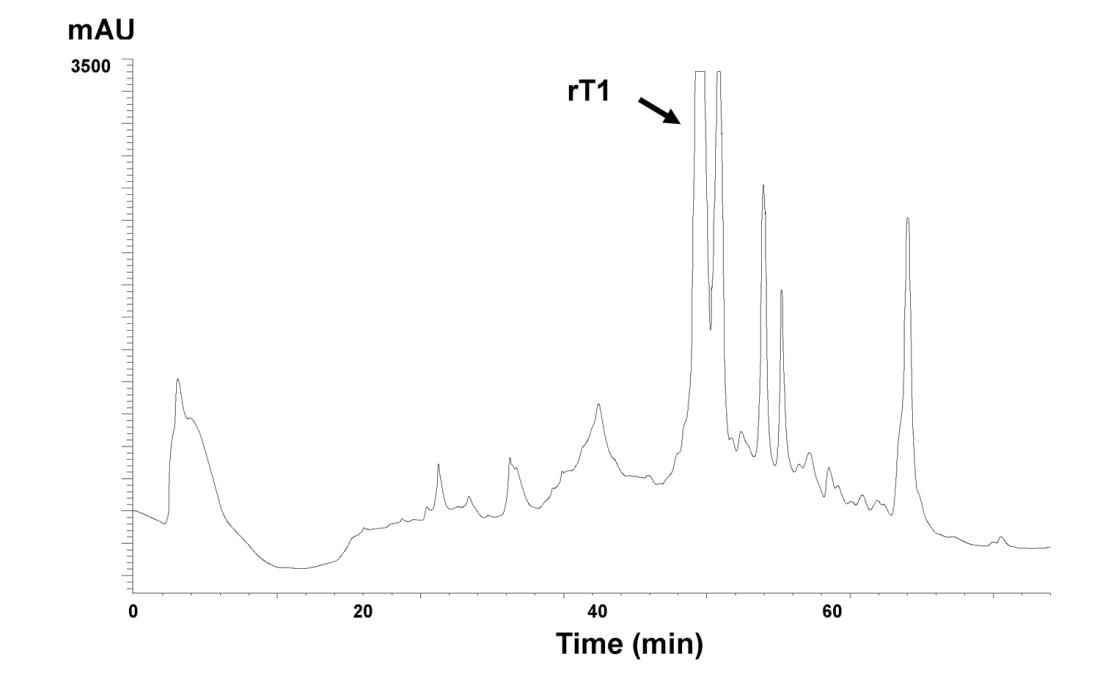


**Supplementary data S2.** HPLC-DAD chromatogram of aqueous extracts of the calyces of *Hibiscus sabdariffa*.

**Supplementary data S3.** *Hibiscus sabdariffa* transcript database search for roseltides using PEAKS software

| **Peptide fragment for roseltide rT1** | **Mass** | **Retention time (min)** |
| --- | --- | --- |
| LSGCCNSPGCIFGICA | 1715.798 | 14.53 |
| SGCCNSPGCIFGICA | 1602.714 | 12.57 |
| VALSGCCNSPGCIFGICA | 1885.903 | 18.12 |
| CIPRGGICLVA | 1186.668 | 15.81 |
| CIPRGGICLVALSGCCNSPGCIFGICA | 2884.455 | 23.47 |
| SPGCIFGICA | 1052.515 | 19.63 |
| GCCNSPGCIFGICA | 1515.682 | 12.67 |
| ALSGCCNSPGCIFGICA | 1786.835 | 15.8 |

| **Peptide fragment for roseltide rT7** | **Mass** | **Retention time (min)** |
| --- | --- | --- |
| CVSSGIVDACSECCEPD | 1887.783 | 8.08 |
| CVSSGIVDACSECCEPDKCIIMLPTWPPRYVCSV | 3960.892 | 24.64 |

**A**

**B**

**Supplementary data S4.** Chemical shift assignment of ^1^H, ^1^H-NOESY spectrum of roseltide rT1. **(A)** The assignments of the NOE cross peaks between side chain protons and amide protons are displayed. **(B)** The assignments of the NOE cross peaks between amide protons are displayed.

*
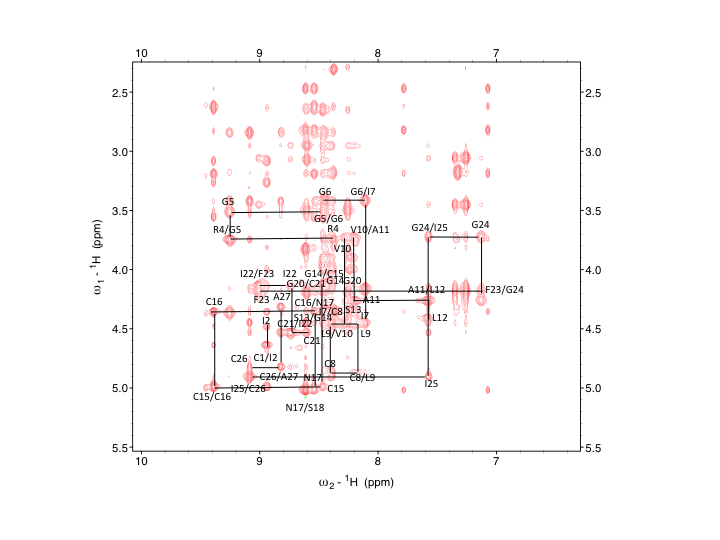
*

**Supplementary data S5.** NOE cross peaks between HN_i_ and Hα_i_, Hαi_-1_.

**A**

**B**

*
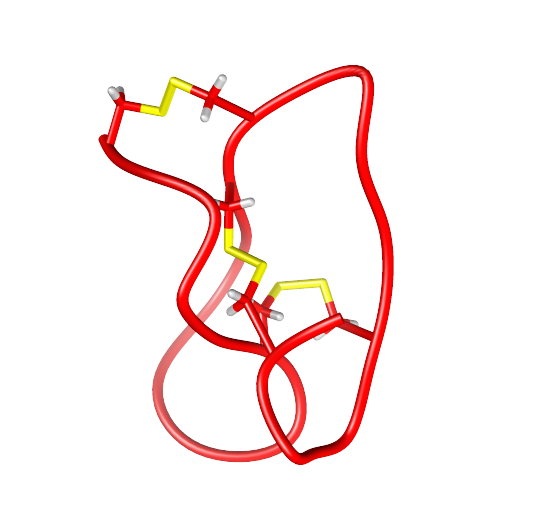
*

*
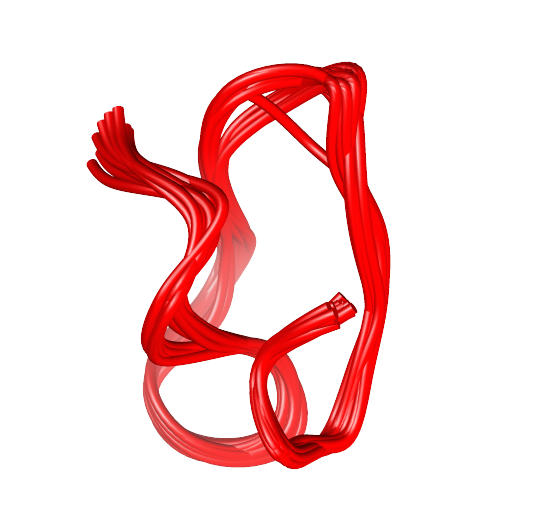
*

N terminus

C terminus

**C**

**D**

C1

C16

C15

C26

C21

C8


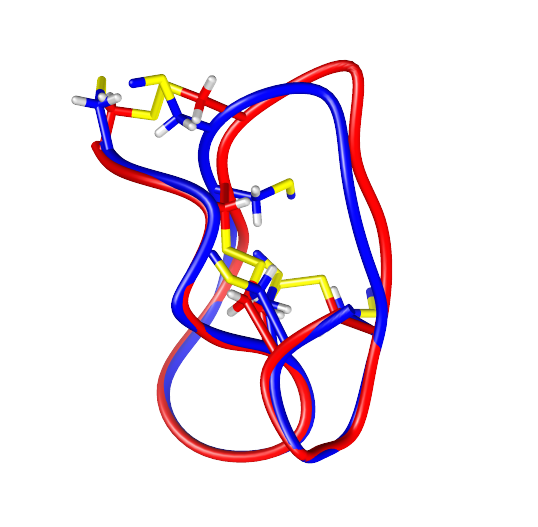
*
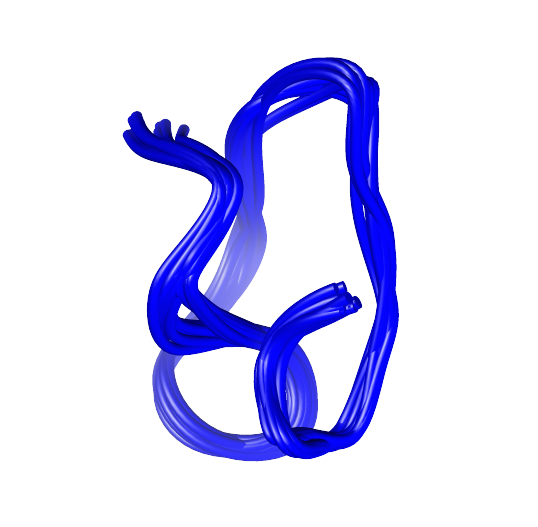
*

N terminus

C terminus

C1

C16

C15

C26

C21

C8

**Supplementary data S6.** Structure and disulfide connection of rT1. **(A)** The 20 best structures generated by CNSsolve 1.3 with the disulfide bonds imposed are displayed in ribbon representation. **(B)** The three disulfide bonds, Cys1-Cys16, Cys8-Cys21 and Cys 15-Cys26, are highlighted in yellow. The proton H_β1_ and H_β2_ are displayed in grey. **(C)** The 20 best structures generated by CNSsolve 1.3 assuming all the cysteines reduced are displayed in ribbon representation. **(D)** The structure generated by CNSsolve 1.3 without disulfide bonds imposed (blue) is superimposed to the structure with disulfide bonds imposed (red): Cys1-Cys16, Cys8-Cys21 and Cys 15-Cys26, in which the six cysteines are reduced and oxidized respectively.

**Supplementary data S7.** Chemical shifts of the protons of roseltide rT1 determined using ^1^H, ^1^H- TOCSY and NOESY.

| Res | HN(ppm) | H_α_(ppm) | | H_β_(ppm) | | Others(ppm) | |
| --- | --- | --- | --- | --- | --- | --- | --- |
| C1 |  | 4.635 |  | 3.259 | 3.078 |  |  |
| I2 | 8.939 | 4.476 |  | 1.887 |  | Hϒ 1.043 | Hδ 0.882 |
| P3 |  | 4.356 |  | 2.305 | 2.01 | Hϒ 1.867 | Hδ 4.006, 3.81 |
| R4 | 8.378 | 3.743 |  | 1.722 | 1.606 | Hϒ 1.487 | Hδ 3.178 Hε 7.327 |
| G5 | 9.256 | 4.366 | 3.504 |  |  |  |  |
| G6 | 8.453 | 4.157 | 3.414 |  |  |  |  |
| I7 | 8.107 | 4.446 |  | 1.774 |  | Hϒ 1.29, 1.000 | Hδ 0.905 |
| C8 | 8.396 | 4.863 |  | 3.182 | 2.957 |  |  |
| L9 | 8.16 | 4.453 |  | 1.663 |  | Hϒ 1.572 |  |
| V10 | 8.284 | 3.728 |  | 2.098 |  | Hϒ 0.926 |  |
| A11 | 8.206 | 4.263 |  | 1.352 |  |  |  |
| L12 | 7.583 | 4.41 |  | 1.666 | 1.579 | Hϒ 0.922 | Hδ 0.872 |
| S13 | 8.241 | 4.345 |  | 3.999 | 3.898 |  |  |
| G14 | 8.425 | 4.133 | 3.968 |  |  |  |  |
| C15 | 8.466 | 4.99 |  | 3.554 | 2.648 |  |  |
| C16 | 9.391 | 4.355 |  | 3.19 | 2.623 |  |  |
| N17 | 8.54 | 5.017 |  | 2.823 | 2.471 |  | Hδ 7.783, 7.073 |
| S18 | 8.613 | 4.449 |  | 3.827 |  |  |  |
| P19 |  | 4.743 |  | 2.29 | 2.178 | Hϒ 1.951, 1.544 | Hδ 3.456 |
| G20 | 8.257 | 4.19 | 3.495 |  |  |  |  |
| C21 | 8.604 | 4.529 |  | 3.067 | 2.957 |  |  |
| I22 | 8.74 | 4.142 |  | 1.98 |  | Hϒ 1.366, 1.174, 0.71 | Hδ 0.628 |
| F23 | 9.009 | 4.179 |  | 3.452 | 3.057 |  | Hδ 7.351, 7.257 |
| G24 | 7.129 | 4.257 | 3.727 |  |  |  |  |
| I25 | 7.574 | 4.901 |  | 1.748 |  | Hϒ 1.433, 1.194 | Hδ 0.826 |
| C26 | 9.085 | 4.823 |  | 3.424 | 2.839 |  |  |
| A27 | 8.822 | 4.313 |  | 1.487 |  |  |  |


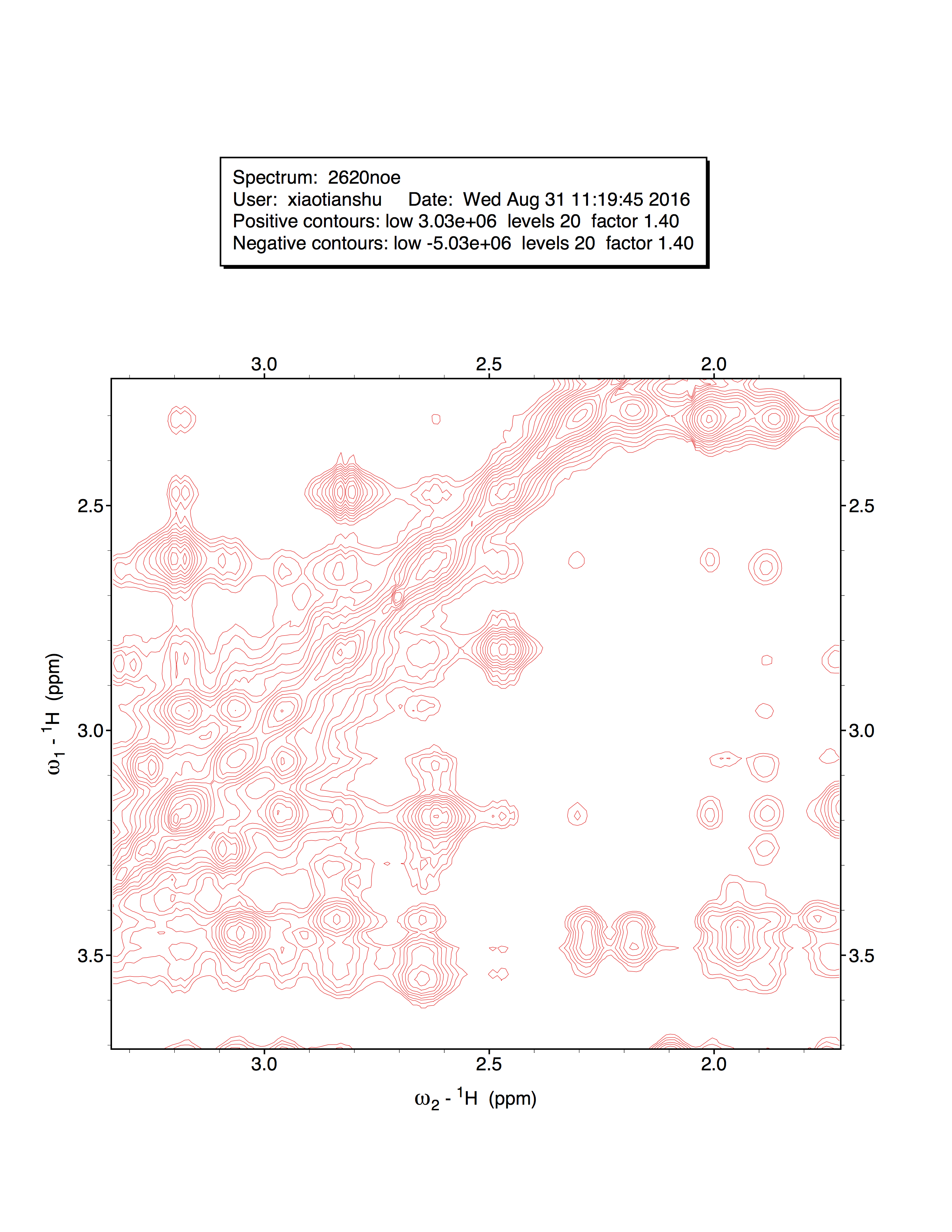


C1 H_β_ /C16 H_β_

C26 H_β_ /C15 H_β_

C21 H_β_ /C8 H_β_

**Supplementary data S8.** NOE cross peak between the Hβs of the two cysteines in each disulfide bond of rT1.

**Supplementary data S9.** Different combinations of disulfide connections and the averaged energies of the 20 best structures generated by CNSsolve 1.3 accordingly.

|  | Disulfide Bond Pattern | * Energy (kcal/mol) |
| --- | --- | --- |
| 0 | No Disulfide Bond Imposed | 465.85±8.32 |
| 1 | CysI-CysIV, CysII-CysV, CysIII-CysVI | 487.06±5.54 |
| 2 | CysI-CysII, CysIV-CysV, CysIII-CysVI | 921.61±9.59 |
| 3 | CysI-CysII, CysIII-CysIV, CysV-CysVI | 781.58±7.83 |
| 4 | CysI-CysII, CysIV-CysVI, CysIII-CysV | 705.96±19.29 |
| 5 | CysI-CysV, CysII-CysIV, CysIII-CysVI | 1057.37±42.08 |
| 6 | CysI-CysV, CysII-CysIII, CysIV-CysVI | 839.72±30.02 |
| 7 | CysI-CysV, CysII-CysVI, CysIV-CysIII | 813.08±16.69 |
| 8 | CysI-CysIII, CysII-CysIV, CysV-CysVI | 792.71±30.87 |
| 9 | CysI-CysIII, CysII-CysV, CysIV-CysVI | 665.80±8.92 |
| 10 | CysI-CysIII, CysII-CysVI, CysIV-CysV | 978.28±122.78 |
| 11 | CysI-CysVI, CysII-CysIII, CysIV-CysV | 825.75±12.01 |
| 12 | CysI-CysVI, CysII-CysIV, CysIII-CysV | 1043.75±44.67 |
| 13 | CysI-CysVI, CysII-CysV, CysIII-CysIV | 773.75±4.43 |
| 14 | CysI-CysIV, CysII-CysIII, CysV-CysVI | 566.61±7.49 |
| 15 | CysI-CysIV, CysII-CysVI, CysIII-CysV | 552.02±12.66 |

*The overall energy is the summary of the energies of bond, angle, dihed, impropers, van der waals (vdw), noe and dihedral angle (cdih).


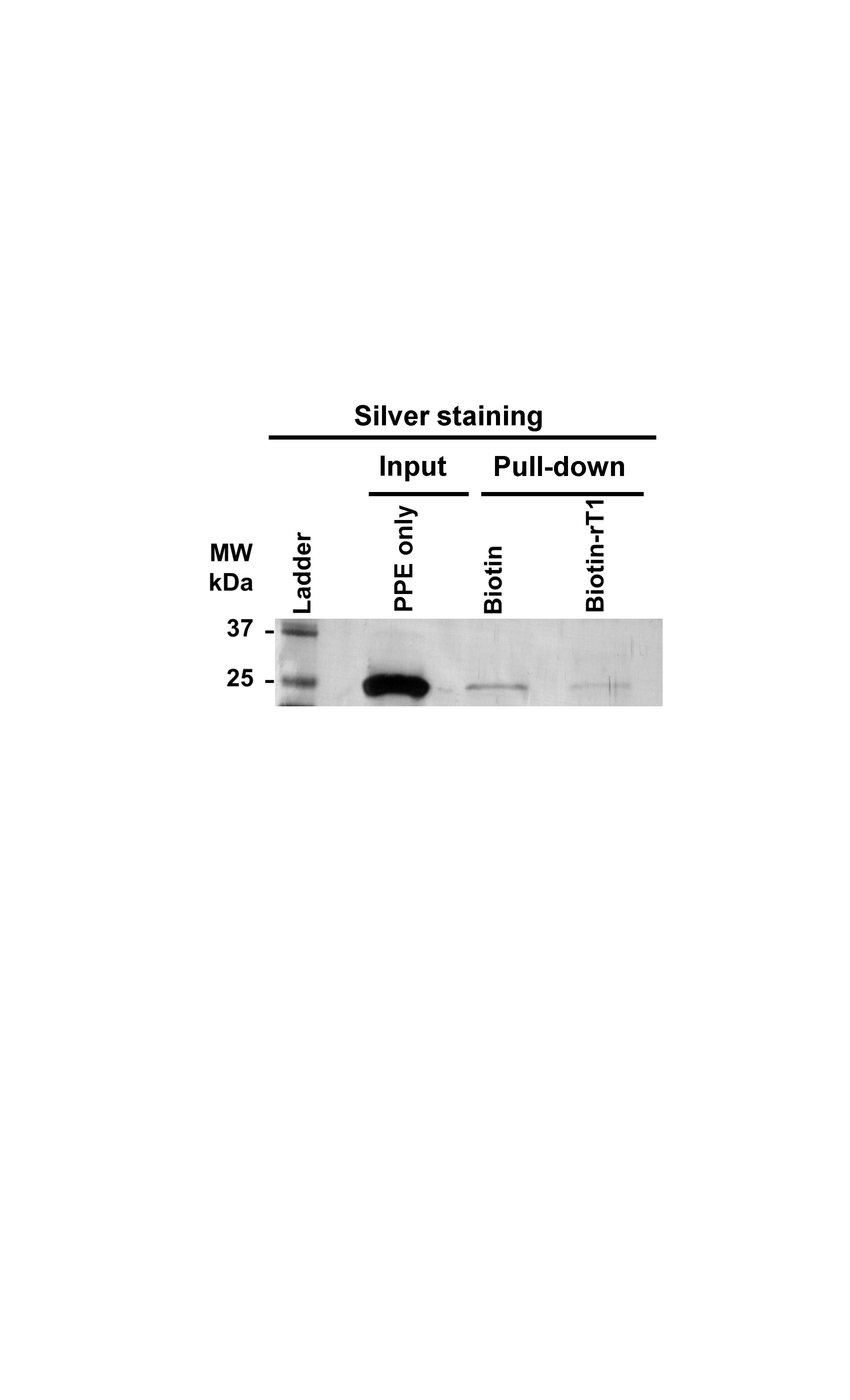


**Supplementary data S10.** SDS-PAGE of the pull-down complex between PPE and biotin-rT1. The leftmost lane is a protein marker (Bio-rad, US). PPE only lane: purified HNE only. Biotin lane: purified PPE incubated with biotin and NeutrAvidin resin (control). Biotin-rT1 lane: purified PPE incubated with biotin-rT1 and NeutrAvidin resin.


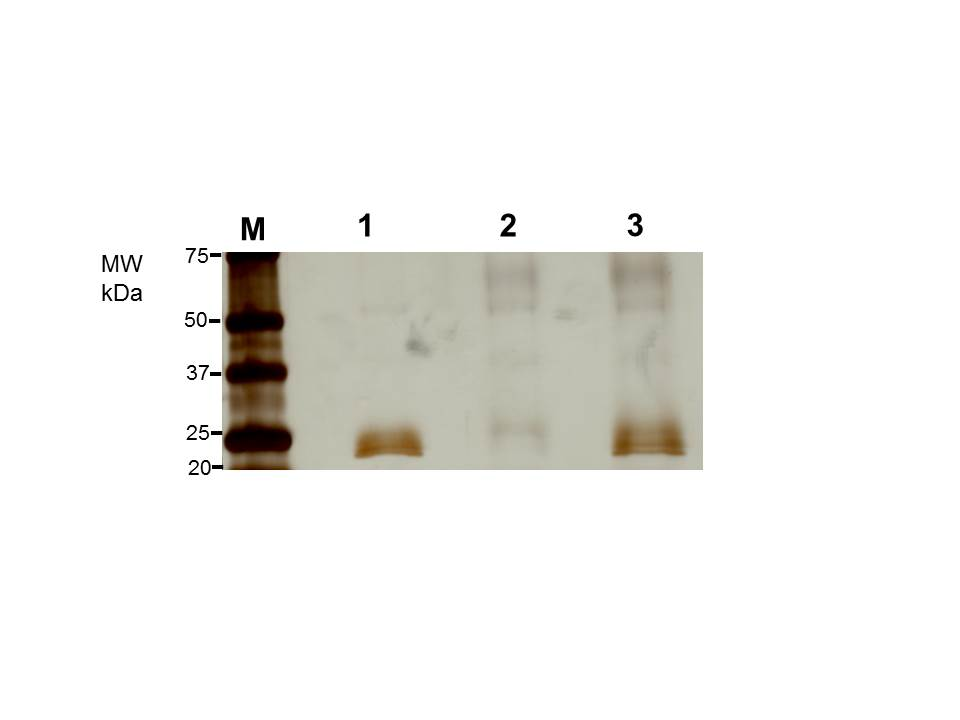


**Supplementary data S11.** Silver-stained SDS-PAGE of the pull-down complex between HNE and biotin-rT1. Lane M shows a protein marker (Bio-rad, US); Lane 1 shows the HNE-only lane: purified HNE only; Lane 2 is the biotin lane: purified HNE incubated with biotin and NeutrAvidin resin (control) and Lane 3 is the biotin-rT1 lane: purified HNE incubated with biotin-rT1 and NeutrAvidin resin.
